# Risk factors for *Echinococcus multilocularis* intestinal infections in owned domestic dogs in a North American metropolis (Calgary, Alberta)

**DOI:** 10.1038/s41598-024-55515-6

**Published:** 2024-03-01

**Authors:** Emilie Toews, Marco Musiani, Anya Smith, Sylvia Checkley, Darcy Visscher, Alessandro Massolo

**Affiliations:** 1 Department of Biological Sciences, University of Calgary, Calgary, AB Canada; 2 School of Public Health, Faculty of Medicine, Imperial College London, London, UK; 3 Department of Biological, Geological and Environmental Sciences (BiGeA), University of Bologna, Bologna, Italy; 4 Faculty of Veterinary Medicine, University of Calgary, Calgary, AB Canada; 5 School of Population and Public Health, Faculty of Medicine, University of British Columbia, Vancouver, BC Canada; 6 Department of Biology, The King’s University, Edmonton, AB Canada; 7 Naturalis Biodiversity Center, Leiden, The Netherlands; 8 Department of Biological Sciences, University of Alberta, Edmonton, AB Canada; 9 Ethology Unit, Department of Biology, University of Pisa, Via Volta 6, 56126 Pisa, Italy; 10 UMR CNRS 6249 Chrono-Environnement, Université Franche-Comté, Besançon, France; 11 BC Centre for Disease Control, Vancouver, BC Canada

**Keywords:** Alveolar echinococcosis, Intestinal echinococcosis, Domestic dogs, *Echinococcus multilocularis*, Canada, Cross-sectional study, Diseases, Risk factors

## Abstract

Human alveolar echinococcosis is increasingly documented in Alberta, Canada. Its causative agent, *Echinococcus multilocularis* (*Em*), can be transmitted to humans by infected dogs. We assessed the prevalence and associated risk factors for *Em* infections in domestic dogs in Calgary, Alberta, Canada. In this cross-sectional study that coupled collection and assessment of dog feces with a survey on potential risk factors, 13 of 696 (Bayesian true prevalence, 2.4%; 95% CrI: 1.3–4.0%) individual dogs’ feces collected during August and September 2012 were qPCR positive for *Em*. Sequencing two of these cases indicated that both were from the same *Em* European strain responsible for human infections in Alberta. Likelihood of intestinal *Em* was 5.6-times higher in hounds than other breeds, 4.6-times higher in dogs leashed at dog parks than those allowed off-leash, 3.1-times higher in dogs often kept in the backyard during spring and summer months than those rarely in the yard, and 3.3-times higher in dogs living in neighbourhoods bordering Bowmont park than those in other areas of Calgary. This situation warrants surveillance of dog infections as a preventative measure to reduce infections in North America.

## Introduction

Infections of dogs by *Echinococcus multilocularis* (*Em* hereafter)—a tapeworm of the Northern Hemisphere^1^—have been increasing in Europe and Asia, but have seldom been reported in North America e.g.^2,3^. In previous reports, *Em* occurred predominantly in rural areas^2,3^, where there are abundant definitive hosts (e.g., dogs, coyotes, foxes, and wolves; DHs hereafter)^4^ and intermediate hosts (rodents and some lagomorphs; IHs hereafter)^4^. However, wild DHs commonly inhabit urban and suburban areas, bringing zoonotic diseases into cities^5,6^. Wild DHs occur in much lower numbers than domestic dogs in these areas, but once an *Em* lifecycle has been established, dogs can perpetuate and maintain this parasite in urban habitats^7,8^. Dogs may have a lower worm burden than their wild counterparts, but individual worms that infect dogs seem to shed more eggs than when infecting coyotes or foxes^9^, and adult worms may actually persist longer in dogs^10^, causing all these DHs to have similar biotic potential. Therefore, it is possible that dogs in metropolitan areas such as Calgary, Alberta (Canada), with a dog population exceeding 135,000 (2016 census data), could be paramount in maintaining an urban *Em* population^11^.

Moreover, not only can dogs act as proficient components of the urban *Em* lifecycle, but they also may transmit the parasite to humans, resulting in human alveolar echinococcosis (AE, hereafter)^12^, a disease of extreme importance in Europe^13^ and worldwide^12^. Although AE is listed as food-borne^12^, dog ownership may be an even greater risk factor for human AE^14^. Whereas 91% of global human AE cases occur in China^15^, a recent and unprecedented surge of cases has been reported in Alberta, where these infections were previously never reported^16–18^. Importantly, genotyping parasitic material from hepatic lesions of these patients indicated infections by a haplotype (labeled “ECA”) sharing more genetic similarity to European *Em* than to the strain endemic to North America^17^. This ECA haplotype was also responsible for most of the recent *Em* infections in Alberta wildlife^17^, perhaps due to differential virulence among strains^19^.

Intrinsic and extrinsic factors may influence the probability of intestinal echinococcosis in domestic dogs. Pooled odds ratios revealed that dogs that are rural, free-roaming, or used for hunting are at higher risk for intestinal *Em* infection^2^. In rural areas of China, guard dogs were more commonly infected than dogs with other uses^20^. Two studies also reported male dogs were infected more often than female dogs^11,21^. Lastly, dogs frequently fed livestock offal were more likely to be infected by *Echinococcus* spp.^21^. Other risk factors have also been investigated, e.g., age, free-roaming range size, time spent walking in rural areas^2^; however, results were inconclusive. To our knowledge, an analysis of the relationship between likelihood of intestinal *Em* infection and dog breed and breed-related behaviours has not been attempted in published literature.

The goal of this study was to investigate risk factors for intestinal infections by *Em* in domestic dogs from a large metropolitan area in Alberta, where an unprecedented cluster of human AE cases occurred. Specifically, we aimed to: (1) estimate prevalence of intestinal *Em* in owned dogs living near city dog parks in Calgary, AB, Canada; (2) assess possible intrinsic and extrinsic risk factors for *Em* infection in owned dogs in this setting; and finally (3) characterize the *Em* strain infecting Calgary dogs, comparing it to the one associated with the recent surge in human cases in AB, Canada.

## Materials and methods

### Study area

Calgary (51°50′N, 114°55′W), is a metropolitan city of > 1.4 million people^22^, which sprawls over 5098 km^2^ in the southern Alberta grasslands in Canada^22^. Elevation ranges from 1060 to 1240 m asl, with two river valleys and several creeks and water bodies providing much riparian habitat^23^. Calgary parks, green spaces, and golf courses house much urban wildlife, including wild canids (coyote and red fox; *Canis latrans* and *Vulpes vulpes,* respectively) and various rodent species that are potential IHs for *Em*^24^. The climate is highland continental, entailing long, variable winters and short, warm summers with average daily temperatures from − 6.8 °C (19.8 °F) in December to 16.5 °C (61.7 °F) in July (climate.weather.gc.ca).

In 2016, the Calgary dog population (135,070) had increased by 12,745 dogs since 2010 and more than doubled in the previous decade^25^. Dog-ownership across Calgary ranged from one dog for every five to seven households in the small city center to one dog for every two or three households in the southwest and southeast quadrants (2016 civic census data). Stray dogs were not present in the City.

### Sampling design

**Figure 1:**
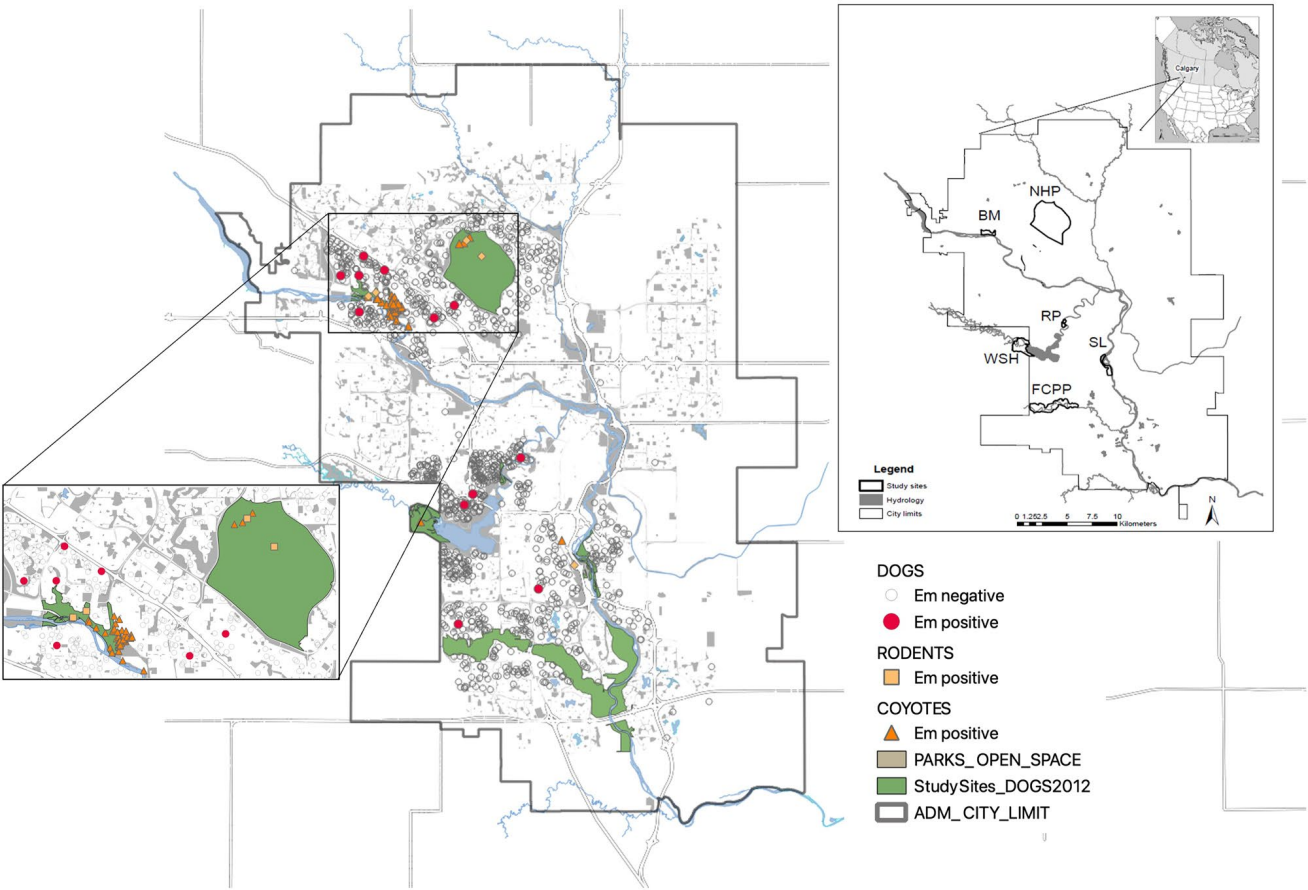
Location of animals infected with *Echinococcus multilocularis* in Calgary, Alberta, Canada in 2012. Dog location (DOGS; hollow circles, *Em* negative dogs; red circles, *Em* positive dogs) is their place of residence. Dogs were sampled from communities bordering six city parks: Bowmont Park (BM), Southland Lowlands (SL), Nosehill Park (NHP), Weaselhead Flats (WSH), Fish Creek Provincial Park (FCPP), and River Park (RP). Coyote location (COYOTES *Em* positive; orange triangles) is the site of *Em* positive fecal sample collection as detailed in Liccioli et al.^24^. Rodent location (RODENTS *Em* positive; light orange squares) is the trap site of *Em* positive rodents detailed in Liccioli et al.^24^. Coyotes and rodents were sampled in all parks and communities bordering all parks except RP^24^.

Our target population was owned dogs living in communities directly bordering (explained below) any of six city parks: River Park (RP), Nosehill Park (NHP), Fish Creek Provincial Park (FCPP), Weaselhead Flats (WSH), Bowmont Park (BM), and Southland Lowlands (SL)^26^ (Fig. 1).

Participants were recruited in a previous study^26^ by randomly selecting 6000 dog owners from the City of Calgary’s 2011 dog license database, including 1000 living in residential communities bordering each of these parks. In June 2012, selected dog owners were sent a recruitment letter via ground mail by the Animal Services and Bylaw Division of the City of Calgary, which contained a website address and password for the online survey as outlined in Smith et al.^26^. Individuals were excluded from the original parasitological analysis if the survey was incomplete, or a fecal sample was not supplied. In the present study, individuals were excluded if there was not enough fecal sample available (2 g) for DNA extraction.

### Survey design

All selected dog-owners were asked to complete a survey including 25 questions organized in seven sections (Supplementary Material 1). In total, 1293 respondents completed the survey, 1082 of which agreed to sample collection^26^. At 222 residences where the individual agreed to collection, no fecal sample was provided, resulting in a total of 860 fecal samples being collected from dog-owner residences during two 2-day collection periods occurring in August and then September 2012^26^.

### Sample processing

Prior to processing, all samples were frozen at − 80 °C for 72 h to inactivate *Em* eggs^27^ in 2012 and then stored at − 20 °C until the present study started in 2018. Of 860 fecal samples, 696 had enough feces left for testing within this study. Molecular methods are detailed further in Supplementary Material 2.

### Dog demographics assessment

We performed a descriptive analysis of survey data to describe the distribution of potential risk factors in the sample. Kruskal–Wallis tests were used to analyze differences in Likert-scaled responses on walking behaviors during the spring and summer months (beginning of May to end of August) such as the amount of time spent by dogs in various outdoor environments (e.g., dog parks, sidewalks and streets, school and sports fields) and the proportion of time dogs spent off-leash in these environments. Dog breeds, as reported by their owner, were categorized into classes based on Canadian Kennel Club (CKC) standards^28^ unless they were of mixed-breed, in which case they were classified as “mixed”. Exact Chi-square tests were used to detect differences in intrinsic (e.g., breed, sex) and extrinsic characteristics (e.g., time spent walking in dog parks and other areas, time spent off-leash in these areas, time spent in the backyard). Throughout the text, means are reported along with their standard error (SEM), and medians with the interquartile ranges (mdn, [IQR]).

### Assessing risk factors

We analyzed various risk factors that could be associated with *Em* positivity using Pearson Chi-squared goodness-of-fit tests^29^, Mantel–Haenszel adjusted odds ratios^30^, and single-variable binary logistic regression i.e., logit model^31^. Specifically, the extrinsic variables tested included: time spent walking in city parks and off-leash in these parks, time spent alone in the yard, and known coyote *Em* prevalence in 2012 and 2013^24^ in the closest park. Intrinsic factors such as frequency of rodent predation, number of dogs in each household, dog breed, and sex were also analyzed. Odds ratios and their corresponding 95% Confidence Intervals (CI) were obtained for all significant risk factors to determine likelihood of *Em* infection for dogs with these intrinsic and extrinsic characteristics. Odds ratios and CIs were gleaned directly from the logistic regression with a logit link for numeric and ordinal data, but were estimated separately post hoc for categorical data.

### True prevalence estimates

True prevalence of *Em* in dogs—overall, and also surrounding each park—was determined to account for the analytic specificity estimate (100%, inputted into the model as 97.5–99.9% based on the very high specificity of the test) and the 95% confidence interval of the sensitivity (70.2–96.4%) of the qPCR^32^ that were used in a Bayesian prevalence model where we implemented the sensitivity and specificity distributions using two chains containing 10,000 “burn-in” samples and 10,000 samples that were retained^33^. For comparison, we also calculated the true prevalence of *Em* infections in both coyotes and rodents in each Calgary park using data from previous studies^24^. Bayesian true prevalence values are accompanied by 2.5 and 97.5% credible intervals (CrI) provided by the model. Statistical analyses were performed in *SPSS v.25* (IBM^®^, Armonk, NY, US), and using the package ‘*prevalence*’ in R Software version 4.0.2 (2020-06-22) to estimate Bayesian true prevalence.

The Canadian Council on Animal Care's (CCAC) guidelines were followed, and the study was approved by the Veterinary Sciences Animal Care Committee (ACC Study permits: #AC14-0075; #AC17-0147) and by the Research Ethics Board (REB Certifications: #REB15-2721; #REB18-1471) of the University of Calgary.

### Ethical standards

The authors confirm that the ethical policies of the journal, as noted on the journal’s author guidelines page, have been adhered to and the appropriate animal care and ethical review committee approvals have been received. The study is reported in accordance with ARRIVE guidelines (https://arriveguidelines.org).

## Results

### Sample characteristics

Between one and seven dogs were owned per household sampled (median: 1, IQR: 1–2). Almost all dogs were spayed or neutered (667/695, 96.0%) and male and female dogs occurred equally (350/692, 50.6% and 342/692, 49.4% respectively) (Table 1). Ages ranged from pups under 1 year old to senior dogs of 17 years old (mean: 7.0 ± 0.1 years). Most dogs were purebred (434/694; 62.5%) rather than of mixed breed (261/695; 37.5%) (*χ*^2^ = 43.1, df = 1, *p* < 0.0001) and the most common pure-breeds were Labrador retriever (57/694; 8.2%), terrier (general) (41/694; 5.9%), golden retriever (28/694; 4.0%), and bichon frise, border collie, and shi-tzu (19/694 each; 2.7% each). Of the purebred dogs, most (257/440; 58.4%) were reported to be breeds with high-prey drive regarding rodents (*χ*^2^ = 12.4, df = 1, *p* < 0.001), based on descriptions of dog breeds by the CKC^28^. Specifically, most pure-breed dogs in this sample were of sporting breeds compared to all other breed classes (*χ*^2^ = 65.2, df = 6, *p* < 0.001) and the proportion of dogs belonging to each class (including sporting, hound, working, terrier, toy, non-sporting, herding, and mixed) was similar in communities surrounding each city park (*χ*^2^ = 42.1, df = 35, *p* = 0.2) (Table 1).

**Table 1:** Intrinsic factors describing dogs sampled around six Calgary (Alberta) city parks (WSH, SL, RP, NHP, FCPP, BM) in 2012 that were screened for intestinal *Echinococcus multilocularis* infections*.*

Park areas	Age (%)			Sex (%)		Spayed or neutered (%)		CKC breed class (%)							
	Pup (< 1 y)	Adult (3–8 y)	Senior (> 8 y)	Male	Female	Yes	No	Sporting	Hound	Working	Terrier	Toy	Non-sporting	Herding	Mixed
WSH	1.3	57.0	41.6	51.4	48.7	96.6	3.4	20.1	5.4	8.1	10.7	6.7	8.7	9.4	30.9
SL	1.4	66.7	31.9	58.3	41.7	97.2	2.8	18.1	5.6	9.7	9.7	12.5	6.9	2.8	34.7
RP	0.9	57.4	41.7	55.7	44.4	95.7	4.4	19.1	9.6	9.6	11.3	7.8	4.3	5.2	33.0
NHP	2.4	72.2	25.4	39.7	60.3	96.8	3.2	13.4	3.9	7.1	14.2	6.3	5.5	7.1	42.5
FCPP	0.0	69.8	30.2	56.5	43.5	96.5	3.5	11.6	7.0	4.7	7.0	14.0	15.1	5.8	34.9
BM	2.7	66.7	30.6	48.0	52.1	94.6	5.4	16.3	2.7	4.1	9.5	6.1	11.6	8.2	41.5
All	1.6	64.5	34.0	50.6	49.4	96.1	3.9	16.7	5.5	7.0	10.6	8.2	8.6	6.9	36.5

*CKC* Canadian Kennel Club, *BM* Bowmont Park, *FCPP* Fish Creek Provincial Park, *NHP* Nosehill Park, *RP* River Park, *SL* Southland Lowlands, *WSH* Weaselhead Flats.

Activity levels of dogs were also similar across sample locations (*χ*^2^ = 19.7, df = 20, *p* = 0.5). In most cases, dogs were moderately to often kept in the yard rather than in the house while on the property from the beginning of May to end of August each year (603/692; 87.1%) (Table 2). Overall, when away from the property, dogs were more frequently walked in dog parks (*χ*^2^ = 824.2, df = 4, *p* < 0.0001), followed closely by on sidewalks and streets (Table 2). However, dogs sampled near FCPP were more frequently walked on sidewalks and streets than in dog parks (Table 2), visiting parks less often than dogs living at other sampling locations (*H* = 16.0, *df* = 5, *p* = 0.007).

**Table 2:** The percentage of time during the beginning of May to end of August in 2012 that was spent in the yard instead of the house by dogs sampled around Calgary (Alberta) parks, and the percentage of time these dogs were walked in other locations, as reported by Smith et al*.*^26^.

Park areas	Time spent in yard (%)			Park	Area most often frequented outside the yard (%)				
	Never or rarely	Moderately	Often		Sidewalks/streets	School/sport fields	Mountains	None	Acreage
WSH	11.5	74.3	14.2	55.8	37.4	0.0	0.0	6.1	0.7
SL	2.8	83.3	13.9	47.1	38.2	2.9	0.0	10.3	1.5
RP	12.3	78.9	8.8	56.1	34.6	0.9	2.8	5.6	0.0
NHP	9.6	73.6	16.8	49.2	37.7	1.6	3.3	6.6	1.6
FCPP	11.6	73.3	15.1	39.0	40.2	3.7	3.7	12.2	1.2
BM	15.0	72.8	12.2	53.6	37.9	2.1	1.4	4.3	0.7
All	11.1	75.4	13.4	51.2	37.5	1.7	1.8	6.9	0.9

*WSH* Weaselhead Park, *SL* Southland Lowlands, *RP* River Park, *NHP* Nosehill Park, *FCPP* Fish Creek Provincial Park, *BM* Bowmont Park.

### *Echinococcus multilocularis* positive dogs and their characteristics

In total, 13 of 696 dog fecal samples tested positive for the *nad2* gene of *Em* by qPCR. The cycle of quantification (Ct) value for these samples ranged from 27.79–37.82 (average 34 ± 0.7).

The 13 positive dogs consisted of four neutered males and nine spayed females between 2 and 14 years (average 7 ± 1 years) (Table 3). Most (7/13; 53%) were from single-dog households although six participants recorded owning two or more dogs. Eight (61%) of the infected dogs were purebred, whereas five (38%) were of mixed breed (Table 3). Of the eight purebred dogs infected by *Em*, seven (88%) were of breeds with high prey drive^28^. The infection distribution across breeds in purebred dogs deviated slightly from the overall sample distribution (χ^2^ = 12.5, df = 6, *p* = 0.047), with infections occurring in sporting, hound, terrier, and non-sporting breed classes (Table 3). No sign of *Em* infection was found in purebred working, toy, or herding breed classes.

**Table 3:** Characteristics of dogs living near Calgary (Alberta) city parks that tested positive for intestinal *Echinococcus multilocularis* infection during a cross-sectional study from July to September 2012.

Park areas^a^	Breed (CKC breed class number or mixed)^b^	High prey drive breed	Sex	Age (y)	Area most-walked	Epg^c^
BM	Bichon frise (6)	Yes	Female	6	Sidewalk/street	3.8
BM	German shepherd/boxer (X)	Unknown^d^	Female	2	nd	12.5
BM	Labradoodle (X)	Unknown	Female	3	Dog park	4.8
BM	Labrador/shepherd (X)	Unknown	Female	3	Sidewalk/street	5.0
BM	Bichon frise (6)	Yes	Female	10	Dog park	2.4
BM	Golden retriever (1)	No	Female	2	Sidewalk/street	2.5
FCPP	German shepherd/Belgian Malinois (X)	Unknown	Male	7	Dog park	10.0
NHP	Collie/terrier (X)	Unknown	Female	8	Mountains	2.5
RP	Miniature dachshund (2)	Yes	Male	14	Dog park	16.0
RP	Terrier (any) (4)	Yes	Male	9	Sidewalk/street	14.3
SL	Basset hound (2)	Yes	Female	5	Dog park	0.9
WSH	Redbone coonhound (2)	Yes	Male	11	Sidewalk/street	2.6
WSH	Kerry blue terrier (4)	Yes	Female	6	Sidewalk/street	19.1

*CKC* Canadian Kennel Club, *nd* no data supplied by participant.

^a^Park areas include: Bowmont Park (BM), Fish Creek Provincial Park (FCPP), Nosehill Park (NHP), RP (River Park), Southland Lowlands (SL), and Weaselhead Flats (WSH); ^b^1 = sporting, 2 = hound, 3 = working, 4 = terrier, 5 = toy, 6 = non-sporting, 7 = herding, X = mixed; ^c^Epg: eggs per gram of fecal sample; ^d^Prey drive could not be determined for mixed-breed dogs.

Dogs that had intestinal echinococcosis were mostly walked on sidewalks and streets (6/13; 46%) or in dog parks (5/13; 38%), except for one dog that was more frequently walked in the mountains and one dog for which no data were available (Table 3). Infected dogs were walked in dog parks approximately two to six times per week (mdn, [IQR]; 5, [3–6]) and similarly on sidewalks and streets (5, [4–6]). These dogs were almost never walked in other areas and walking off-leash rarely occurred in any of these areas (Table 3).

### True prevalence estimates of *E*. *multilocularis*

The Bayesian true prevalence of *Em* in dogs living around Calgary dog parks was 2.4% (95% CrI: 1.3–4.0%), after accounting for qPCR sensitivity and sensitivity. The true prevalence of *Em* in hounds (12.2%; CrI: 3.4–26.2%) was two-fold higher than in non-sporting breeds (6.1%; CrI: 1.3–14.4%), which was the next most highly affected breed class. There was no difference in *Em* prevalence between mixed and purebred dogs (χ^2^ = 0.005, df = 1, *p* = 1.00), although there is some evidence for infection prevalence being higher in hounds than in other purebred classes χ^2^ = 12.5, df = 6, *p* = 0.047.

**Figure 2:**
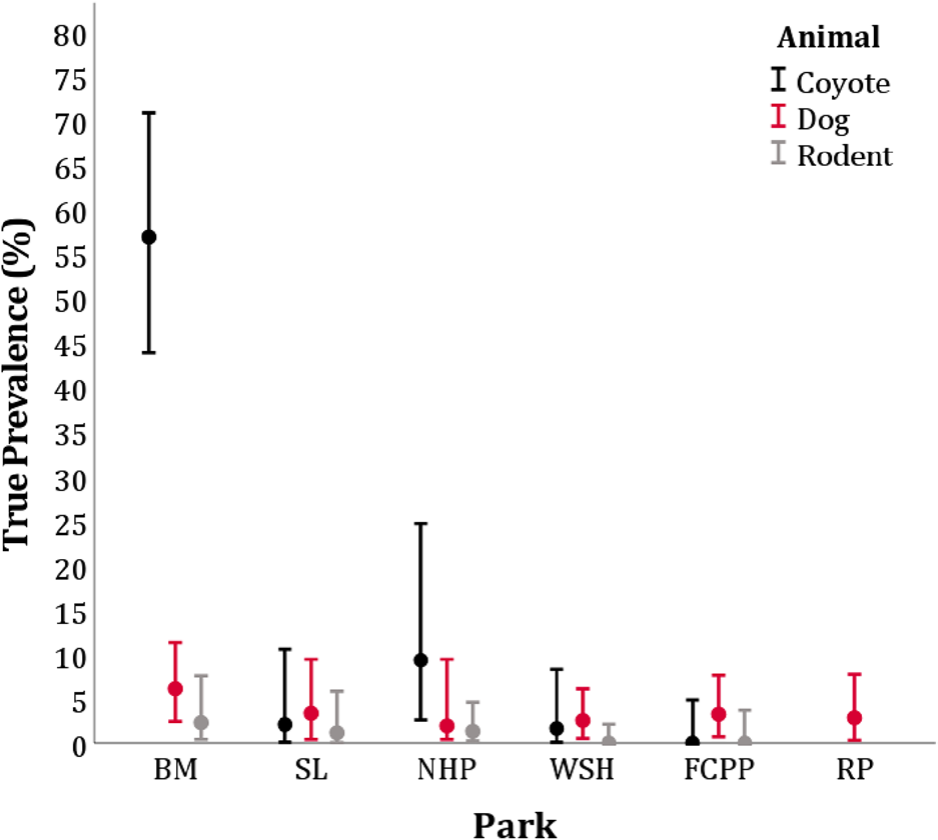
Bayesian true prevalence (and 95% credible intervals) of *Echinococcus multilocularis* in dogs, coyotes, and rodents in Calgary, Alberta, Canada in 2012. Dogs were sampled in postal codes adjacent to six city parks: Bowmont (BM), Southland Lowlands (SL), Nosehill Park (NHP), Weaselhead Flats (WSH), Fish Creek Provincial Park (FCPP), and River Park (RP). Coyotes and rodents were sampled in and around all parks except RP. Coyote and rodent Bayesian true prevalence values were estimated using data from Liccioli et al.^24^.

The Bayesian true prevalence in previously sampled coyotes was 16.2% (95% CrI: 12.0–20.7%) with a significantly high prevalence of infection recorded in BM and NHP^24^ (Fig. 2). For rodents sampled in the same study, the Bayesian *Em* true prevalence was calculated to be 1.0% (95% CrI: 0.4–1.9%) with a higher prevalence again occurring in BM, although this difference was not found to be significant due to the low number of positive cases^24^ (Fig. 2).

### European-type strain genotyping

Microscopy of egg sediment retrieved from the ZnCl_2_ flotation/sedimentation analysis indicated that all 13 positive dogs were actively shedding Taeniid species eggs at sample collection. These dogs were shedding between 0.9 and 19.1 eggs per gram of feces (median: 7.2, [2.5–12.9]) (Table 3). Seven to nine eggs were isolated per fecal sample, except for one sample where only one egg was obtained. Thus, a total of 97 single Taeniid eggs were isolated from the 13 samples. The *nad1* gene was successfully amplified in a total of 15 eggs from seven of the 13 samples. Viable sequences were obtained only for two of the 15 eggs which came from different samples. Both sequences were identical to the *Em* haplotype E (KF962559), a European-like haplotype previously described in coyotes and a dog from central British Columbia, Canada^34^.

### Risk factors for infections

#### Intrinsic factors

**Figure 3:**
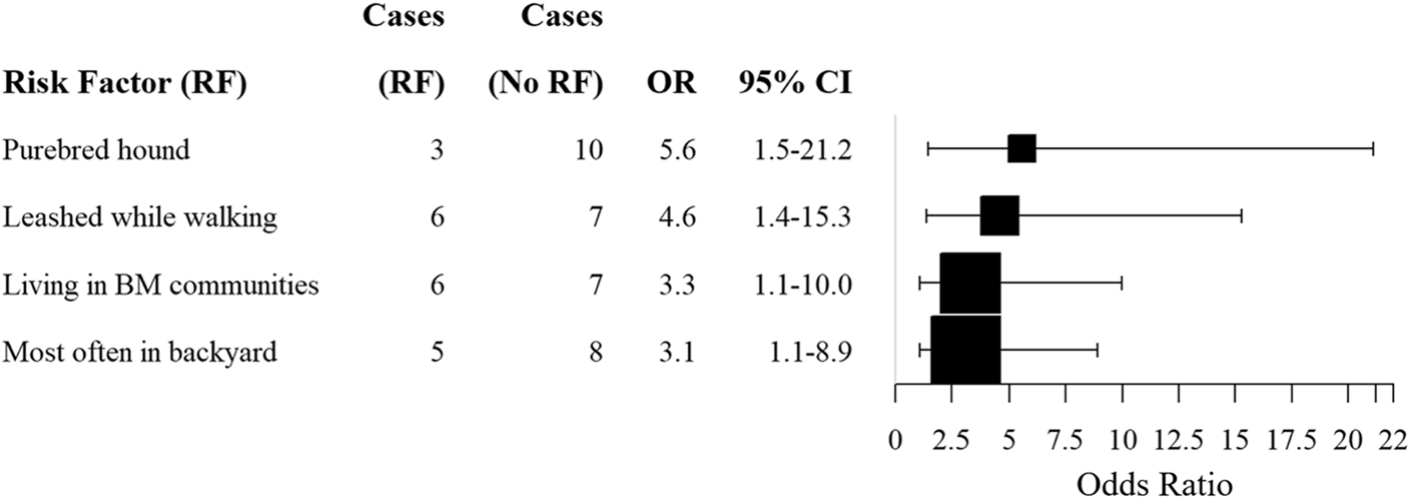
Odds ratios (OR) with associated 95% confidence intervals for each risk factor that had a significant relationship (OR > 1) with presence of intestinal *Echinococcus multilocularis* infection in domestic dogs sampled in communities surrounding six Calgary city parks (Bowmont (BM), Southland Lowlands (SL), Nosehill Park (NHP), Weaselhead Flats (WSH), Fish Creek Provincial Park (FCPP), and River Park (RP)) in 2021. Box size was scaled to the number of infected dogs with the associated risk factor.

Only one intrinsic risk factor was significantly associated with the likelihood of dog infection with *Em*. Purebred hounds were 5.6 times more likely (95% CI: 1.5–21.1; Fig. 3) to carry intestinal *Em* infections than all other breeds, including mixed breeds (*χ*^2^ = 8.0, df = 1, *p* = 0.029), and 6.8 times more likely (95% CI: 1.6–29.8) to be infected than other pure breeds (*χ*^2^ = 5.2, df = 1, *p* = 0.023). No other breed class showed a significant association with the probability of *Em* intestinal infection.

#### Extrinsic factors

Extrinsic factors were also associated with the probability of infection with intestinal *Em*. First, dogs kept on-leash at dog parks were 4.6 times (95% CI: 1.4–15.3) more likely to be infected with intestinal echinococcosis (z = 2.5, *p* = 0.01; Fig. 3). As well, a high proportion (5/13; 38.5%) of infected dogs were most often kept in a yard when at home (*χ*^2^ = 7.1, df = 2, *p* = 0.03). Compared to dogs that were rarely or never kept in a yard at home during the spring and summer months, dogs that were most often kept in the yard were 3.1 times more at risk than those that were rarely or never kept in the yard (95% CI: 1.1–8.9; Fig. 3).

#### Spatial factors

Almost half (6/13; 46.2%) of the infected dogs lived near Bowmont Park (BM) (Fig. 1). When comparing the proportion of infected BM dogs to those living in all other sampled areas, more BM dogs were infected than all other dogs (*χ*^2^ = 5.0, df = 1, *p* = 0.03) (Fig. 2) and BM dogs were 3.3 (95% CI: 1.1–10.0) times more likely to be infected than dogs living near other city parks.

## Discussion

To the knowledge of the authors, only four studies have been published on the prevalence of *E. multilocularis* in domestic dogs in Canada^3,35–37^, though only one detected evidence of infection^3^. However, in the present study, the prevalence in dogs living around dog parks in Calgary, Alberta, resembled reported estimates from Eastern Europe and Asia^2^. Additionally, parasite eggs recovered in this study that were genotyped appeared to belong to a European-like strain of *Em*^34^, although only two viable sequences could be obtained from the 15 eggs retrieved.

In regard of risk factors, no previous *Em* study has ever estimated the level of *Em* likelihood of infection associated with specific dog breeds. In our study, purebred hounds seemed to have a significantly higher likelihood of intestinal infection by *Em* than other dog breeds, including mixed-breed, possibly due to their historically human-selected behavioral traits. Over 400 dog breeds currently exist and are distinguished by varying appearance and behavior^38^. This large number of distinguishable breeds developed due to selective breeding so that dogs could fulfill certain functions and achieving standards like those set by kennel clubs, e.g., the CKC^39^. Specifically, hounds were bred for independent hunting and for flushing and catching rodents^38^ and hunting behavior remains an intrinsic trait of these breeds^40^. It is therefore likely that this study’s hounds captured and consumed more rodents and had a higher per capita rate of exposure to *Em* through infected prey. Previous studies on other gastrointestinal parasites have been in frequent disagreement about whether purebred dogs are more likely to be parasitized than mixed breeds^41^ or vice versa^42^, whether likelihood of infection is dependent on the dog breed and type of parasite^43^, or whether the difference in infection levels among breeds is actually absent^44–47^. However, it is also important to note that many studies, including this one, rely on owner-reporting of breed based on appearance, which may not always be accurate, especially for dogs of mixed-breed^48,49^.

As for walking habits, unexpectedly, we determined that dogs were more likely to be infected with intestinal *Em* when they were always kept on-leash at city dog parks, although this result is limited by the univariable analysis and does not provide additional insight into the relationship between leashing tendencies and other walking habits. In a previous study, dogs kept more frequently off-leash were more likely to be infected with *Toxocara canis*—which can employ a similar route of transmission to *Em*^50^. Similarly, park-attending dogs that were frequently off-leash when walked were more likely to be parasitized by *Giardia* species^26^. The discrepancy with previous findings could perhaps be explained by the fact that hounds, the most infected breed class in this study, are both a rodent-hunting breed class, and are also known to be less trainable to follow owners off-leash^40^. It is however plausible that such dogs were in contact with rodents in other contexts than off-leash areas in parks, e.g., while in home yards.

Although several studies on gastrointestinal parasitism in dogs reported a positive correlation between park attendance and likelihood of infection^26,51–53^, our study seemed to imply otherwise, possibly due to our focus on *Em*. Remarkably, *Em* infection was more highly associated with time spent in the yard at home, although univariable analysis hindered further insight into associations between time spent in the yard and other behaviours. A dog roaming its yard freely and unsupervised may have opportunities to hunt small mammals (e.g., mice or voles) on the property. Dogs that prey upon rodents are 2.9 times more likely to be parasitized by endoparasites^54^; therefore, the prolonged opportunity for preying on rodents in the yard could increase *Em* transmission to dogs.

In our study the neighborhood’s environment was one of the most important risk factors for *Em* infections in dogs. Dogs sampled around Bowmont Park (BM) had a significantly higher prevalence of *Em* than those living around other parks (Fig. 3), even though demographics were constant across groups. This could be due to the concurrent high prevalence of the parasite in wild DHs and IHs, including coyotes and rodents, respectively, around BM, and the relatively high proportion of IH species compared with species not acting as IHs in the area^24^. Our findings, therefore, supported the notion that urban wildlife can be source of infections in humans and dogs^54,55^.

### Limitations and conclusions

Acknowledging that our sample was not representative of the overall Calgary dog population (we surveyed dogs from communities around parks for which we had estimates of *Em* prevalence in wild hosts), if we apply our prevalence estimate to the overall dog population in Calgary (135,070 pet dogs, 2016), we may conservatively expect that up to a few thousand dogs have been shedding infectious eggs through their feces in as many households, since the sample collection originally occurred in 2012. This could have increased the likelihood of ingestion of parasite egg by dog owners via multiple routes: directly, by petting or handling dog hair where eggs have attached^56,57^; indirectly, through defecation of eggs into vegetable gardens^58^, or by transfer to the household^7,8,58^.

However, we have thus far only summarized the *Em* situation of Calgary in 2012, and due to the recent increase in human AE numbers across Alberta^16,17^, intestinal infections in domestic dogs need to be more thoroughly studied and updated, perhaps through a surveillance system. Moreover, although we could genotype only two samples out of 13, likely due to both the degradation of the DNA in the samples which were stored at − 20 °C for five years and the difficulties in strain-typing eggs of Taeniidae species, attention should nonetheless be paid to the strain of *Em* that was detected in the two samples that could be genotyped. Actually, although the number of genotyped samples was likely not representative of the situation, the fact that the sequenced eggs more closely resembled the strains endemic to Europe should warrant caution, especially considering that EU-like strains have been more recently described in wildlife^59^ and, more worryingly, in all the human cases of AE that have been strain-typed so far in Alberta (5 out of 7)^17,18^. Notably, all strain-typed human cases^17,18^ were caused by the ECA strain, which was the most prevalent (78.1%, 75/96 cases) in wild definitive hosts (coyotes and foxes) sampled in Alberta between 2012 to 2017^60^.

Moreover, despite the absence of the North American haplotype in dogs sampled in our study could be due to low sample size of strain-typed eggs, a more reasonable explanation could be its low occurrence in wildlife hosts (around 2–3%)^59,60^ and/or to a suggested lower infectivity than the European-like haplotypes^17^. More broadly, urbanization and the encroachment of residential areas upon wild landscapes provides ample opportunity for parasites like *Em* to take advantage of new routes of transmission provided by the increase in urban-adapted wildlife hosts^6^, particularly considering that a European-like strain, likely originated from an invasion process^60^, is now circulating in both wild and domestic DHs. For these reasons and due to its transmissibility to and morbidity in humans, *Em* is an emerging infectious disease whose risk factors deserve uttermost attention^18,61^.

### Supplementary Information

Supplementary Information 1.

Supplementary Information 2.
