# Supplementary material for: Risk factors for Echinococcus multilocularis intestinal infections in owned domestic dogs in a North American metropolis (Calgary, Alberta)
Source: Sci Rep. 2024 Mar 1;14:5066. doi: 10.1038/s41598-024-55515-6 (PMC10907371; doi:10.1038/s41598-024-55515-6)
Supplement: Supplementary file 1 — Supplementary Information 1. [file 41598_2024_55515_MOESM1_ESM.docx]

Toews et al.: Risk factors for Echinococcus multilocularis intestinal infections in owned domestic dogs in a North American metropolis (Calgary, Alberta)

# **SUPPLEMENTARY MATERIAL 1: Dog behaviour questionnaire distributed to dog owners who consented to participate in the pilot study**

**Calgary Urban Dog Study**

OBJECTIVES & INCENTIVES: My name is Anya Smith, I am a PhD student (under the supervision of Dr. Alessandro Massolo) conducting a study on gastrointestinal parasites (microorganisms affecting the intestines) in park attending and non park-attending dogs living in the vicinity of six popular recreation areas in Calgary. I am asking for your help because our research team wants to understand gastrointestinal parasitism in these urban dogs. Our ultimate goals are to protect animal health as well as the public’s health.

BACKGROUND: any gastrointestinal parasites will only infect dogs or coyotes, but there is a small risk that some could also be transmitted to humans. That is why this study is relevant for both dog and human health. We want to understand what people can do to minimize the risk of infection for dogs and transmission to humans. That is why our survey asks about dog-care, including dog-walking. We will share the results of this study with park and wildlife managers, public health experts, veterinarians, and dog owners like yourself.

WHAT WILL I BE ASKED TO DO? We ask that you complete this 15-minute survey in full. There are no right or wrong answers. We simply want to understand what you do to take care of your dog, especially where and how frequently your dog is taken for walks. At the end of this survey, we ask if you are willing to provide a fecal sample from your dog at a later date. If you agree to this, and are selected from the pool of willing participants, we will send you a labeled bag with instructions for sample collection (we will pick the sample up from your residence, please see below for further details).WHAT HAPPENS TO THE INFORMATION I PROVIDE?Your participation is voluntary, and you can withdraw from the study at any point. All or part of your information may be used as data in Anya Smith's thesis report, journal publication(s), conference presentations, posters, and future research that will be carried out within Dr. Massolo’s laboratory at the Faculty of Veterinary Medicine. Dog and owner identities will remain anonymous and confidential in all of the above described uses for the data. We will provide the results of your dog’s fecal analysis to you only if it is positive (in which case, the results from your dog's lab analysis will not be confidential to the researchers, but will remain so in the above described uses for the data). Hardcopies of the survey, transcribed reports, and results of fecal analyses will be stored in a secure, lockable cabinet at the University. Any electronic versions of dog and owner personal data, and fecal analysis results will be kept on the password-protected computer hard-drive of my computer or servers belonging to the University of Calgary. Only approved members of the research team will have access to this material. If you opt to withdraw from the study, the fecal sample and any partially completed data will be retained. Your participation in this survey will imply consent.

HOW COULD PARTICIPATING IN THIS STUDY BE BENEFICIAL FOR ME AND MY DOG? Those who agree to provide a fecal sample from their dog will be entered into the pool of participants who could be contacted for a follow-up study. If you are contacted, we will be asking you to provide a fecal sample from your dog and would mail you a labeled Ziploc bag in which to place the sample. We would collect this sample from your residence, all you would need to do is leave the sample outside your door in the bag, on a specified collection date. If your dog tests positive for one of the parasites we are investigating, we will use the information provided in the survey to contact you. In addition, we will provide a report on this study to all dog-owners who are willing to provide a sample of their dog's feces and who provide an email address.**Please note that all questions in this survey must be answered to be eligible for lab analysis.** Thank you very much for participating. Please feel free to contact us with any questions. Anya Smith & Alessandro Massolo: (email) urbandog@ucalgary.ca; (p) 403-210-7405.Please note that you must be over the age of 18 years and one of the owners of the dog described in this survey to participate.

|  | I AGREE |
| --- | --- |

# Are you over the age of 18 years?

|  | Yes |
| --- | --- |
|  | No |

# Are you the owner of the dog who you will be describing in this survey?

*Please note: if you own more than one dog, please select only one as the focus of this survey.

|  | Yes |
| --- | --- |
|  | No |

# What is the name of your residential community?

|  | Acadia |
| --- | --- |
|  | Altadore |
|  | Bayview |
|  | Beddington Heights |
|  | Bel-aire |
|  | Bowness |
|  | Brentwood |
|  | Brittania |
|  | Cambrian Heights |
|  | Canyon Meadows |
|  | ... 22 additional choices hidden ... |
|  | Riverbend |
|  | Sandstone |
|  | Scenic Acres |
|  | Shawnee Slopes |
|  | Silver Springs |
|  | Thorncliffe |
|  | Varsity Estates |
|  | Willow Park |
|  | Woodbine |
|  | Woodlands |

# PART 1: QUESTIONS ABOUT YOUR DOG.

# What is the age of your dog?

|  | 1 year or less |
| --- | --- |
|  | Greater than 1 year to 2 years |
|  | Greater than 2 years to 3 years |
|  | Greater than 3 years to 4 years |
|  | Greater than 4 years to 5 years |
|  | Greater than 5 years to 6 years |
|  | Greater than 6 years to 7 years |
|  | Greater than 7 years to 8 years |
|  | Greater than 8 years to 9 years |
|  | Greater than 9 years to 10 years |
|  | ... 4 additional choices hidden ... |
|  | Greater than 15 years to 16 years |
|  | Greater than 16 years to 17 years |
|  | Greater than 17 years to 18 years |
|  | Greater than 18 years to 19 years |
|  | Greater than 19 years to 20 years |
|  | Greater than 20 years to 21 years |
|  | Greater than 21 years to 22 years |
|  | Greater than 22 years to 23 years |
|  | Greater than 23 years to 24 years |
|  | Greater than 24 years to 25 years |

# What gender is your dog?

|  | MALE |
| --- | --- |
|  | FEMALE |

# What breed(s) is your dog?

|  | Other |
| --- | --- |
|  | Cross or Mixed Breed |
|  |  |
|  | Akita |
|  | Alaskan Malamute |
|  | American Bulldog |
|  | American or Canadian Eskimo |
|  | Australian Cattle Dog |
|  | Australian Shepard |
|  | Basenji |
|  | ... 26 additional choices hidden ... |
|  | Papillon |
|  | Pomeranian |
|  | Pug |
|  | Rottweiler |
|  | Shetland Sheep Dog |
|  | Shih Tzu |
|  | Siberian Husky |
|  | Standard Poodle |
|  | Terrier |
|  | Toy Poodle |

# Please specify your dog's breed(s), to the best of your ability:

# Is your dog neutered or spayed?

|  | YES |
| --- | --- |
|  | NO |
|  | UNKNOWN |

# Approximately how much does your dog weigh?

Please answer in pounds or kilograms.

| Pounds | \|  \| UNKNOWN \| \| --- \| --- \| \|  \| 1 \| \|  \| 2 \| \|  \| 3 \| \|  \| 4 \| \|  \| 5 \| \|  \| 6 \| \|  \| 7 \| \|  \| 8 \| \|  \| 9 \| \|  \| ... 280 additional choices hidden ... \| \|  \| 291 \| \|  \| 292 \| \|  \| 293 \| \|  \| 294 \| \|  \| 295 \| \|  \| 296 \| \|  \| 297 \| \|  \| 298 \| \|  \| 299 \| \|  \| 300 \| |
| --- | --- | --- | --- | --- | --- | --- | --- | --- | --- | --- | --- | --- | --- | --- | --- | --- | --- | --- | --- | --- | --- | --- | --- | --- | --- | --- | --- | --- | --- | --- | --- | --- | --- | --- | --- | --- | --- | --- | --- | --- | --- | --- | --- |
| Kilograms | \|  \| UNKNOWN \| \| --- \| --- \| \|  \| 1 \| \|  \| 2 \| \|  \| 3 \| \|  \| 4 \| \|  \| 5 \| \|  \| 6 \| \|  \| 7 \| \|  \| 8 \| \|  \| 9 \| \|  \| ... 280 additional choices hidden ... \| \|  \| 291 \| \|  \| 292 \| \|  \| 293 \| \|  \| 294 \| \|  \| 295 \| \|  \| 296 \| \|  \| 297 \| \|  \| 298 \| \|  \| 299 \| \|  \| 300 \| |

# What type of diet do you provide your dog?

|  | KIBBLE |
| --- | --- |
|  | RAW FOOD |
|  | OTHER |
|  | COMBINATION |

# Please specify what diet you provide your dog:

Please specify the form(s) of food you provide your dog, not the brand(s) of food you provide.

# PART 1: DOG INFORMATION CONTINUED.

# How many dogs reside in your household?

|  | 1 |
| --- | --- |
|  | 2 |
|  | 3 |
|  | 4 |
|  | 5 |
|  | 6 |
|  | 7 |
|  | 8 |
|  | 9 |
|  | 10 |
|  | ... 180 additional choices hidden ... |
|  | 192 |
|  | 193 |
|  | 194 |
|  | 195 |
|  | 196 |
|  | 197 |
|  | 198 |
|  | 199 |
|  | 200 |
|  | > 200 |

# Approximately how many times,if at all, has your dog visited the veterinarian in the last 6 months?

|  | 0 |
| --- | --- |
|  | 1 |
|  | 2 |
|  | 3 |
|  | 4 |
|  | 5 |
|  | 6 |
|  | 7 |
|  | 8 |
|  | 9 |
|  | ... 82 additional choices hidden ... |
|  | 93 |
|  | 94 |
|  | 95 |
|  | 96 |
|  | 97 |
|  | 98 |
|  | 99 |
|  | 100 |
|  | > 100 |
|  | UNKNOWN |

# Please summarize the reason(s) why your dog has seen the veterinarian in the last 6 months.

# PART 2: DOG & OWNER ACTIVITIES.

FOR THE NEXT SERIES OF QUESTIONS please include responses ofTYPICAL DOG-WALKING BEHAVIOUR IN THE SPRING AND SUMMER MONTHS ONLY FROM THE BEGINNING OF MAY to THE END OF AUGUST.** Please do not consider the use of streets/sidewalks as a corridor to a park, school yard, or sports field destination as part of time spent walking along streets/sidewalks.

# Where do you spend the most time with your dog outside of the house?

|  | PARK |
| --- | --- |
|  | SIDEWALKS/STREETS |
|  | SCHOOL YARDS/SPORTS FIELDS |
|  | MOUNTAINS |
|  | I DO NOT USUALLY TAKE MY DOG OFF OF MY PROPERTY |
|  | OTHER |
|  |  |

# Please specify where you spend the most time with your dog outside of the house.

# How often, if at all, do you engage in the following activities with your dog?

|  | NEVER | LESS THAN ONCE A MONTH | 1-3 DAYS A MONTH | ONCE A WEEK | 2-6 DAYS A WEEK | 1 OR MORE TIMES PER DAY |
| --- | --- | --- | --- | --- | --- | --- |
| ATTENDING PARKS |  |  |  |  |  |  |
| WALKING ALONG NEIGHBORHOOD SIDEWALKS/STREETS |  |  |  |  |  |  |
| WALKING IN SCHOOLS OR SPORTSFIELDS |  |  |  |  |  |  |
| HUNTING |  |  |  |  |  |  |
| MOUNTAIN ACTIVITIES OTHER THAN HUNTING (HIKING, FISHING, ETC) |  |  |  |  |  |  |
| OTHER |  |  |  |  |  |  |

# If you selected "other" in the last question, please specify the activity/activities. If you have more than one "other" activity, please list in order from the most commonly performed to least commonly performed activities.

# While engaging in the following activities, how much time, if at all, does your dog spend off-leash?

Please indicate time spent off-leash for only those activities that you and your dog participate in.If you indicated "other" in the last question, and you have more than one "other" activity, please specifiy your dog's level of off-leash activity for the most common "other" activity only.

|  | ALL OF THE TIME | MOST OF THE TIME | SOME OF THE TIME | RARELY | NEVER |
| --- | --- | --- | --- | --- | --- |
| ATTENDING PARKS |  |  |  |  |  |
| WALKING ALONG NEIGHBORHOOD SIDEWALKS/STREETS |  |  |  |  |  |
| WALKING IN SCHOOLS OR SPORTSFIELDS |  |  |  |  |  |
| MOUNTAIN ACTIVITIES OTHER THAN HUNTING |  |  |  |  |  |
| OTHER |  |  |  |  |  |

# If you have a fenced backyard, in a usual week, how often is your dog in your backyard?

|  | NEVER |
| --- | --- |
|  | RARELY |
|  | SOMETIMES |
|  | REGULARLY |
|  | ALWAYS |
|  | I DON'T HAVE A FENCED BACKYARD |

# If your dog defecates in your backyard, in a usual week, approximately how many times do you clean dog litter from your backyard?

If your dog does not defecate in your backyard, please skip this question.

|  | 0 |
| --- | --- |
|  | 1 |
|  | 2 |
|  | 3 |
|  | 4 |
|  | 5 |
|  | 6 |
|  | 7 |
|  | 8 |
|  | 9 |
|  | ... 11 additional choices hidden ... |
|  | 22 |
|  | 23 |
|  | 24 |
|  | 25 |
|  | 26 |
|  | 27 |
|  | 28 |
|  | 29 |
|  | 30 |
|  | > 30 |

# In a usual week, does your dog:

|  | NEVER | RARELY | SOMETIMES | REGULARLY | ALWAYS |
| --- | --- | --- | --- | --- | --- |
| SLEEP/REST ON YOUR BED |  |  |  |  |  |
| SLEEP/REST ON COUCH |  |  |  |  |  |
| LICK OWNER(S) FACE(S)/LIPS |  |  |  |  |  |

# Do you allow your dog access to the following rooms?

|  | NEVER | RARELY | SOMETIMES | REGULARLY | ALWAYS |
| --- | --- | --- | --- | --- | --- |
| BEDROOM |  |  |  |  |  |
| KITCHEN |  |  |  |  |  |
| LIVING ROOM |  |  |  |  |  |

# PART 3: PARK ACTIVITY.

FOR THE NEXT SERIES OF QUESTIONS please include responses of TYPICAL /AVERAGE DOG-WALKING BEHAVIOUR IN THE SPRING AND SUMMER MONTHS ONLY FROM THE BEGINNING OF MAY to THE END OF AUGUST.** For our purposes, a park is defined as a natural park (natural vegetation without sports fields) with or without a water body that does not classify as a schoolyard, sports field, urban corridor (a path with green strip on either side in a neighborhood) urban park (a small park with benches or statue as focal point in a neighborhood). Examples of parks include: neighborhood, community, provincial parks, and mixed-use parks (natural park combined with sports field), and off-leash dog parks.

# In a usual week, how many times, if at all, does your dog ATTEND ONE OR MORE PARKS with you or someone else?

|  | 0 |
| --- | --- |
|  | 1 |
|  | 2 |
|  | 3 |
|  | 4 |
|  | 5 |
|  | 6 |
|  | 7 |
|  | 8 |
|  | 9 |
|  | ... 11 additional choices hidden ... |
|  | 22 |
|  | 23 |
|  | 24 |
|  | 25 |
|  | 26 |
|  | 27 |
|  | 28 |
|  | 29 |
|  | 30 |
|  | > 30 |

# To which park is your dog taken most often (please provide the name of one primary park only)?

We would prefer a name, but if you do not know the name of the park, please provide the nearest intersection or street.

# In a usual week, approximately how many times do you visit your PRIMARY PARK?

|  | 1 |
| --- | --- |
|  | 2 |
|  | 3 |
|  | 4 |
|  | 5 |
|  | 6 |
|  | 7 |
|  | 8 |
|  | 9 |
|  | 10 |
|  | ... 10 additional choices hidden ... |
|  | 22 |
|  | 23 |
|  | 24 |
|  | 25 |
|  | 26 |
|  | 27 |
|  | 28 |
|  | 29 |
|  | 30 |
|  | >30 |

# Approximately how much time do you spend at your primary park during a typical visit with your dog?

| MINUTES | \|  \| 1 \| \| --- \| --- \| \|  \| 2 \| \|  \| 3 \| \|  \| 4 \| \|  \| 5 \| \|  \| 6 \| \|  \| 7 \| \|  \| 8 \| \|  \| 9 \| \|  \| 10 \| \|  \| ... 160 additional choices hidden ... \| \|  \| 172 \| \|  \| 173 \| \|  \| 174 \| \|  \| 175 \| \|  \| 176 \| \|  \| 177 \| \|  \| 178 \| \|  \| 179 \| \|  \| 180 \| \|  \| > 180 \| |
| --- | --- | --- | --- | --- | --- | --- | --- | --- | --- | --- | --- | --- | --- | --- | --- | --- | --- | --- | --- | --- | --- | --- | --- | --- | --- | --- | --- | --- | --- | --- | --- | --- | --- | --- | --- | --- | --- | --- | --- | --- | --- | --- | --- |

# During a typical visit, how much time, if at all, does your dog spend off-leash at your primary park?

|  | ALL OF THE TIME |
| --- | --- |
|  | MOST OF THE TIME |
|  | SOME OF THE TIME |
|  | RARELY |
|  | NEVER |

# During a typical visit, how much time, if at all, does your dog spend swimming or wading at your primary park?

|  | ALL OF THE TIME |
| --- | --- |
|  | MOST OF THE TIME |
|  | SOME OF THE TIME |
|  | RARELY |
|  | NEVER |

# During a typical visit, how often, if at all, does your dog catch live mice and/or eat or play with dead mice at your primary park?

|  | ALL OF THE TIME |
| --- | --- |
|  | MOST OF THE TIME |
|  | SOME OF THE TIME |
|  | RARELY |
|  | NEVER |
|  | UNKNOWN |

# PART 3: PARK ACTIVITY CONTINUED.

# Do you visit more than one park on a regular basis (ONCE A WEEK OR MORE)?

|  | Yes |
| --- | --- |
|  | No |

# How many other parks do you visit once a week or more (OTHER THAN YOUR PRIMARY PARK)?

|  | 1 |
| --- | --- |
|  | 2 |
|  | 3 |
|  | 4 |
|  | 5 |
|  | 6 |
|  | 7 |
|  | 8 |
|  | 9 |
|  | 10 |
|  | 11 |
|  | 12 |
|  | 13 |
|  | 14 |
|  | 15 |
|  | 16 |
|  | 17 |
|  | 18 |
|  | 19 |
|  | 20 |
|  | > 20 |

# In a usual week, how many times do you visit other parks, in total?

|  | 1 |
| --- | --- |
|  | 2 |
|  | 3 |
|  | 4 |
|  | 5 |
|  | 6 |
|  | 7 |
|  | 8 |
|  | 9 |
|  | 10 |
|  | ... 10 additional choices hidden ... |
|  | 22 |
|  | 23 |
|  | 24 |
|  | 25 |
|  | 26 |
|  | 27 |
|  | 28 |
|  | 29 |
|  | 30 |
|  | > 30 |

# In a usual week, approximately how much time do you spend in total at other parks?

| MINUTES/WEEK | \|  \| 1 \| \| --- \| --- \| \|  \| 2 \| \|  \| 3 \| \|  \| 4 \| \|  \| 5 \| \|  \| 6 \| \|  \| 7 \| \|  \| 8 \| \|  \| 9 \| \|  \| 10 \| \|  \| ... 1780 additional choices hidden ... \| \|  \| 1792 \| \|  \| 1793 \| \|  \| 1794 \| \|  \| 1795 \| \|  \| 1796 \| \|  \| 1797 \| \|  \| 1798 \| \|  \| 1799 \| \|  \| 1800 \| \|  \| > 1800 \| |
| --- | --- | --- | --- | --- | --- | --- | --- | --- | --- | --- | --- | --- | --- | --- | --- | --- | --- | --- | --- | --- | --- | --- | --- | --- | --- | --- | --- | --- | --- | --- | --- | --- | --- | --- | --- | --- | --- | --- | --- | --- | --- | --- | --- |

# During a typical visit at these other parks, how much time does your dog spend off-leash?

|  | ALL OF THE TIME |
| --- | --- |
|  | MOST OF THE TIME |
|  | SOME OF THE TIME |
|  | RARELY |
|  | NEVER |

# During a typical visit at these other parks, how much time, if at all, does your dog spend swimming or wading at other parks?

|  | ALL OF THE TIME |
| --- | --- |
|  | MOST OF THE TIME |
|  | SOME OF THE TIME |
|  | RARELY |
|  | NEVER |

# During a typical visit at these other parks, how often, if at all, does your dog catch live mice and/or eat or play with dead mice at your primary park?

|  | ALL OF THE TIME |
| --- | --- |
|  | MOST OF THE TIME |
|  | SOME OF THE TIME |
|  | RARELY |
|  | NEVER |
|  | UNKNOWN |

# In a usual week, how many times, if at all do you visit the following parks?

Indicated parks can be your primary park or other parks attended during a usual week.

| RIVER PARK | \|  \| 1 \| \| --- \| --- \| \|  \| 2 \| \|  \| 3 \| \|  \| 4 \| \|  \| 5 \| \|  \| 6 \| \|  \| 7 \| \|  \| 8 \| \|  \| 9 \| \|  \| 10 \| \|  \| ... 10 additional choices hidden ... \| \|  \| 22 \| \|  \| 23 \| \|  \| 24 \| \|  \| 25 \| \|  \| 26 \| \|  \| 27 \| \|  \| 28 \| \|  \| 29 \| \|  \| 30 \| \|  \| > 30 \| |
| --- | --- | --- | --- | --- | --- | --- | --- | --- | --- | --- | --- | --- | --- | --- | --- | --- | --- | --- | --- | --- | --- | --- | --- | --- | --- | --- | --- | --- | --- | --- | --- | --- | --- | --- | --- | --- | --- | --- | --- | --- | --- | --- | --- |
| SOUTHLAND PARK | \|  \| 1 \| \| --- \| --- \| \|  \| 2 \| \|  \| 3 \| \|  \| 4 \| \|  \| 5 \| \|  \| 6 \| \|  \| 7 \| \|  \| 8 \| \|  \| 9 \| \|  \| 10 \| \|  \| ... 10 additional choices hidden ... \| \|  \| 22 \| \|  \| 23 \| \|  \| 24 \| \|  \| 25 \| \|  \| 26 \| \|  \| 27 \| \|  \| 28 \| \|  \| 29 \| \|  \| 30 \| \|  \| > 30 \| |
| NOSEHILL PARK | \|  \| 1 \| \| --- \| --- \| \|  \| 2 \| \|  \| 3 \| \|  \| 4 \| \|  \| 5 \| \|  \| 6 \| \|  \| 7 \| \|  \| 8 \| \|  \| 9 \| \|  \| 10 \| \|  \| ... 10 additional choices hidden ... \| \|  \| 22 \| \|  \| 23 \| \|  \| 24 \| \|  \| 25 \| \|  \| 26 \| \|  \| 27 \| \|  \| 28 \| \|  \| 29 \| \|  \| 30 \| \|  \| > 30 \| |
| BOWMONT PARK | \|  \| 1 \| \| --- \| --- \| \|  \| 2 \| \|  \| 3 \| \|  \| 4 \| \|  \| 5 \| \|  \| 6 \| \|  \| 7 \| \|  \| 8 \| \|  \| 9 \| \|  \| 10 \| \|  \| ... 10 additional choices hidden ... \| \|  \| 22 \| \|  \| 23 \| \|  \| 24 \| \|  \| 25 \| \|  \| 26 \| \|  \| 27 \| \|  \| 28 \| \|  \| 29 \| \|  \| 30 \| \|  \| > 30 \| |
| FISH CREEK PARK | \|  \| 1 \| \| --- \| --- \| \|  \| 2 \| \|  \| 3 \| \|  \| 4 \| \|  \| 5 \| \|  \| 6 \| \|  \| 7 \| \|  \| 8 \| \|  \| 9 \| \|  \| 10 \| \|  \| ... 10 additional choices hidden ... \| \|  \| 22 \| \|  \| 23 \| \|  \| 24 \| \|  \| 25 \| \|  \| 26 \| \|  \| 27 \| \|  \| 28 \| \|  \| 29 \| \|  \| 30 \| \|  \| > 30 \| |
| WEASELHEAD PARK | \|  \| 1 \| \| --- \| --- \| \|  \| 2 \| \|  \| 3 \| \|  \| 4 \| \|  \| 5 \| \|  \| 6 \| \|  \| 7 \| \|  \| 8 \| \|  \| 9 \| \|  \| 10 \| \|  \| ... 10 additional choices hidden ... \| \|  \| 22 \| \|  \| 23 \| \|  \| 24 \| \|  \| 25 \| \|  \| 26 \| \|  \| 27 \| \|  \| 28 \| \|  \| 29 \| \|  \| 30 \| \|  \| > 30 \| |

# PART 3: PARK ACTIVITY CONTINUED.

# What is the primary reason for not attending parks regularly?

|  | PRESENCE OF WILDLIFE |
| --- | --- |
|  | PRESENCE OF DOG LITTER |
|  | PRESENCE OF OFF-LEASH DOGS |
|  | TOO FAR FROM HOME |
|  | OTHER |

# Please specify your reason for not attending parks.

# PART 4: QUESTIONS ABOUT YOU AND YOUR HOUSEHOLD.

Please note that personal information is completely confidential and will be used to determine if social factors are associated to dog parasitism.

# What is your gender?

|  | MALE |
| --- | --- |
|  | FEMALE |
|  | OTHER |

# In what year were you born?

|  | 1910 |
| --- | --- |
|  | 1911 |
|  | 1912 |
|  | 1913 |
|  | 1914 |
|  | 1915 |
|  | 1916 |
|  | 1917 |
|  | 1918 |
|  | 1919 |
|  | ... 64 additional choices hidden ... |
|  | 1985 |
|  | 1986 |
|  | 1987 |
|  | 1988 |
|  | 1989 |
|  | 1990 |
|  | 1991 |
|  | 1992 |
|  | 1993 |
|  | 1994 |

# How many people reside in your household?

|  | 1 |
| --- | --- |
|  | 2 |
|  | 3 |
|  | 4 |
|  | 5 |
|  | 6 |
|  | 7 |
|  | 8 |
|  | 9 |
|  | 10 |
|  | ... 4 additional choices hidden ... |
|  | 16 |
|  | 17 |
|  | 18 |
|  | 19 |
|  | 20 |
|  | 21 |
|  | 22 |
|  | 23 |
|  | 24 |
|  | 25 |

# What is your residential postal code (this information will help us estimate the approximate distance between households and parks)?

# Do you have children 5 years old or under in your household?

|  | Yes |
| --- | --- |
|  | No |

# How many children under 5 years old are in your household?

|  | 1 |
| --- | --- |
|  | 2 |
|  | 3 |
|  | 4 |
|  | 5 |
|  | 6 |
|  | 7 |
|  | 8 |
|  | 9 |
|  | 10 |
|  | 11 |
|  | 12 |
|  | 13 |
|  | 14 |
|  | 15 |
|  | 16 |
|  | 17 |
|  | 18 |
|  | 19 |
|  | 20 |

# PART 4: QUESTIONS ABOUT YOU AND YOUR HOUSEHOLD CONTINUED.

# What is your highest education level completed?

|  | HAVE NOT COMPLETED HIGHSCHOOL |
| --- | --- |
|  | COMPLETED HIGHSCHOOL |
|  | COMPLETED COLLEGE, VOCATIONAL TRAINING |
|  | UNIVERSITY, I.E. UNDERGRADUATE DEGREE |
|  | COMPLETED A GRADUATE DEGREE |
|  | PREFER NOT TO SAY |

# What is your total gross household annual income (including all members of your household except housemates)?

|  | $1000 - 29999 |
| --- | --- |
|  | $30000 - 59999 |
|  | $60000 - 89999 |
|  | $90000 - 119000 |
|  | $120000 - 149999 |
|  | $150000 - 179999 |
|  | $180000 + |
|  | DO NOT KNOW |
|  | PREFER NOT TO SAY |

# Do you have one or more cats in your household?

|  | Yes |
| --- | --- |
|  | No |

# Is your cat:

|  | INDOOR |
| --- | --- |
|  | OUTDOOR |
|  | BOTH |

# What OTHER PETS other than cats or dogs do you have in your household, if any?

|  | LIZARD |
| --- | --- |
|  | TURTLE |
|  | BIRD |
|  | SNAKE |
|  | FISH |
|  | FROG |
|  | HAMSTER |
|  | RAT |
|  | MOUSE |
|  | RABBIT |
|  | FERRET |
|  | OTHER |
|  | NONE |

# Please specify what "other" pet(s) you own.

# PART 5: REQUEST FOR DOG FECAL SAMPLE.

We will be collecting dog feces from select dog owners and analyzing them for Giardia and Cryptosporidium spp. parasites. If the results are positive for one or both parasites,the lab results will be provided to owners at no cost, and will be matched with the data in this survey (all survey and lab data is completely confidential and anonomous in all intended uses for the data - please see page 1 for details). If you are willing to provide a sample of your dog’s feces, we may contact you and send you an information package containing a sample collection bag labeled with a number, and instructions on how to submit the sample (in general, leaving a sample in a labeled bag outside your door on a specified day of the week).**PLEASE COMPLETE THE ENTIRE SURVEY TO BE ELIGIBLE FOR FREE LAB ANALYSIS**

# Would you be willing to provide a sample of your dog’s feces?

|  | Yes |
| --- | --- |
|  | No |

# What is your first and last name?

# What is your dog's name?

# What is your residential address (required for sample pickup)?

| STREET ADDRESS |  |
| --- | --- |
|  |  |
| QUADRANT (NW, NE, SW, OR SE) |  |
| UNIT/SUITE/APT# |  |

# What is your mailing address (please answer only if different from the above residential address. This is required to send an information package)?

| STREET ADDRESS |  |
| --- | --- |
|  |  |
| QUADRANT (NW, NE, SW, OR SE) |  |
| UNIT/SUITE/APT# |  |
| P.O. BOX # |  |

# What is your phone number (in the format ###-###-####)?

# What is your email address (required to forward lab results and a final research report)?

# We will be COLLECTING SAMPLES during the LAST TWO WEEKS OF JULY & AUGUST. Are there any dates that you are aware of that you or a family member would not be able to leave a sample outside (e.g. going on holiday)? If yes, please specify.Please also specify where you will be leaving the sample (e.g. on your front doorway landing, beside the bush at the bottom of the front stairs, at the side of my apartment building on the east side etc). Ideally, please leave the sample in the shade.

# Are you willing to be contacted for future research studies by our team?

|  | Yes |
| --- | --- |
|  | No |

# How would you prefer to be contacted?

|  |  |
| --- | --- |
| PHONE |  |
| EMAIL |  |
| POST |  |

# PART 6: CONTACT INFORMATION

Please fill out the fields applicable to your preferred method of contact.

# What is your first and last name?

# What is your mailing address?

| STREET ADDRESS |  |
| --- | --- |
|  |  |
| QUADRANT (NW, NE, SW, OR SE) |  |
| UNIT/SUITE/APT |  |
| P.O. BOX # |  |

# What is your phone number (in the format ###-###-####)?

# What is your email address?

# A FRIENDLY REMINDER!

# Have you completed all questions asked? To be eligible for lab analysis, please double check that you have answered all questions now by selecting the "BACK" button at the bottom of each page. Pressing "NEXT" will get you back to this page once you are ready to submit your survey.Once you are ready to submit your survey, please click on the "SUBMIT" button at the bottom of this page. Please note that once you have submitted your survey, you will not be able to log back in to change your answers.Thank you very much for participating!
