# Supplementary material for: Risk factors for Echinococcus multilocularis intestinal infections in owned domestic dogs in a North American metropolis (Calgary, Alberta)
Source: Sci Rep. 2024 Mar 1;14:5066. doi: 10.1038/s41598-024-55515-6 (PMC10907371; doi:10.1038/s41598-024-55515-6)
Supplement: Supplementary file 2 — Supplementary Information 2. [file 41598_2024_55515_MOESM2_ESM.docx]

Toews et al.: Risk factors for Echinococcus multilocularis intestinal infections in owned domestic dogs in a North American metropolis (Calgary, Alberta)

# **SUPPLEMENTARY MATERIAL 2:** *Molecular and diagnostics methods*

### DNA extraction

DNA was extracted from 200mg of each fecal sample using the Omega Mag-Bind® Universal Pathogen DNA extraction kit (#M4029-01) as per the manufacturer’s instructions, with one amendment: we added five cycles of freezing with dry ice for 1 minute and heating on a 70°C heat block for 1 minute as outlined in Klein *et al*, 2014 ^2^ between the initial homogenization step and the addition of proteinase K to the sample in order to release the parasite oncosphere from its resilient outer shell ^1^. Automated DNA extraction was performed using a MagMAX^TM^ Express 96 (Applied Biosystems).

### qPCR for detection of E. multilocularis

To detect presence of *E. multilocularis*, a duplex qPCR reaction of the mitochondrial gene *nad2* ^3^ and an internal amplification control (IAC) ^4^ was performed as described in Santa *et al*, 2018 ^3^ using a C1000^TM^ Thermal Cycler Chassis with CFX96^TM^ Optical Reaction Module (Bio-Rad) and visualized using the CFX Maestro^TM^ Software*.* Nad234 primers were used to amplify *E. multilocularis* DNA while IAC was used to assess the presence of PCR inhibitors in the sample. According to previous work, the analytic sensitivity and specificity of this test were 87.1% and 100% respectively ^5^. Samples negative for IAC amplification were diluted using a ten-fold dilution and re-run ^3^ again in duplicate using the same procedure to overcome PCR inhibition.

### Flotation and egg harvesting

Parasite eggs were collected from samples that showed evidence of *E. multilocularis* DNA amplification using ZnCl_2_ flotation on 2g feces ^6^.

We analyzed under a microscope a maximum of three 100μL aliquots of concentrated egg solution collected from the flotation/sedimentation procedure. Once Taeniid eggs were found in an aliquot, all Taeniid eggs on the microscope slide were systematically counted to determine egg density per gram of feces, and no further aliquots were analyzed. Next, 1μL concentrated egg solution was diluted tenfold and ten individual eggs per sample were isolated and harvested under a stereomicroscope as described in Huttner *et al*, 2008 ^7^.

### Nested PCR

Individual Taeniid eggs were lysed in 0.02M NaOH at 95°C for 10 minutes before performing nested PCR on the mitochondrial *nad1* gene using external primers (external forward: TATTAAAAATATTGAGTTTGCGTC, external reverse: TCTTGAAGTTAACAGCATCACGAT) and internal primers (internal forward: TGGAACTCAGTTTGAGCTTTACTA, internal reverse: ATATCAAAGTAACCTGCTATGCAG) ^7^.

The 50μL external reaction contained 25μL AccuStart^TM^ II PCR SuperMix, 1μL each 10μM external primer, 1μL template DNA, and 22μL H_2_O. The 50μL internal reaction was comprised similarly, using internal primers instead of external primers, and 1μL external PCR amplicon instead of template DNA. Each reaction was denatured at 94°C for 2 minutes followed by 35 cycles of denaturation at 94°C for 15 seconds, annealing at 55°C for 30 seconds, and extension at 72°C for 60 seconds in a T100^TM^ Thermal Cycler (Bio-Rad).

Results were visualized on a 3% agarose gel run at 80-150V and post-stained with GelRed® Nucleic Acid Gel Stain (Biotium) for 30 min. Successful nested PCR amplicons were cleaned up using the E.Z.N.A.^®^ Cycle Pure Kit (Omega Bio-tek) prior to strain genotyping.

### Strain genotyping

The whole *nad1* gene (1072bp) was sequenced from single egg DNA amplified by nested PCR. For each reaction, 50 to 100ng of template and 3.2pmol of the internal NAD1 forward primer were diluted with water and sequenced by the University of Calgary Core DNA Services (Calgary, AB). Obtained sequences were uploaded to *Sequence Scanner Software v2.0* (Thermofisher Scientific, Waltham, MA) and assessed for quality. Viable sequences were compared to existing *nad1* templates obtained from GenBank™ (http://www.ncbi.nlm.nih.gov/genbank/). BLAST® (https://blast.ncbi.nlm.nih.gov/Blast.cgi) was used to align the obtained sequences to existing templates.

# **CITED LITERATURE**

1 Veit, P. *et al.* Influence of environmental factors on the infectivity of *Echinococcus multilocularis* eggs. *Parasitology* **110**, 79-86, doi:10.1017/s0031182000081075 (1995).

2 Klein, C., Liccioli, S. & Massolo, A. Egg intensity and freeze-thawing of fecal samples affect sensitivity of Echinococcus multilocularis detection by PCR. *Parasitology Research* **113**, 3867-3873, doi:10.1007/s00436-014-4055-x (2014).

3 Santa, M. A. *et al.* Detecting co-infections of *Echinococcus multilocularis* and *Echinococcus canadensis* in coyotes and red foxes in Alberta, Canada using real-time PCR. *Int. J. Parasitol. Parasites Wildl.*, doi:10.1016/j.ijppaw.2018.03.001 (2018).

4 Deer, D. M., Lampel, K. A. & Gonzalez-Escalona, N. A versatile internal control for use as DNA in real-time PCR and as RNA in real-time reverse transcription PCR assays. *Lett. Appl. Microbiol.* **50**, 366-372 (2010).

5 Santa, M. A., Pastran, S., Klein, C., Ruckstuhl, K. & Massolo, A. Evaluation of an automated magnetic bead-based DNA extraction and real-time PCR in fecal samples as a pre-screening test for detection of *Echinococcus multilocularis* and *Echinococcus canadensis* in coyotes. *Parasitol. Res.* **118**, 119-125, doi:10.1007/s00436-018-6125-y (2019).

6 Liccioli, S., Kutz, S. J., Ruckstuhl, K. E. & Massolo, A. Spatial heterogeneity and temporal variations in Echinococcus multilocularis infections in wild hosts in a North American urban setting. *International Journal for Parasitology* **44**, 457-465, doi:10.1016/j.ijpara.2014.03.007 (2014).

7 Hüttner, M. *et al.* Genetic characterization and phylogenetic position of *Echinococcus felidis* Ortlepp, 1937 (Cestoda: Taeniidae) from the African lion. *Int. J. Parasitol.* **38**, 861-868, doi:10.1016/j.ijpara.2007.10.013 (2008).
